# Aldehyde *dehydrogenase 2* rs671 polymorphism may be associated with an increased risk of coronary atherosclerosis among patients with type 2 diabetes mellitus

**DOI:** 10.3389/fmed.2026.1772461

**Published:** 2026-03-23

**Authors:** Weiyong Xu, Shunfa Wang, Huaqing Yao, Rongtai Luo, Pu Li, Xinping Lan

**Affiliations:** Center for Cardiovascular Diseases, Meizhou People’s Hospital, Meizhou, China

**Keywords:** *ALDH2*, coronary atherosclerosis, risk, susceptibility, type 2 diabetes mellitus

## Abstract

Type 2 diabetes mellitus (T2DM) is a risk for coronary atherosclerosis. However, some T2DM patients are prone to coronary atherosclerosis, while another group do not suffer from it. Are there any risk factors for T2DM patients to develop coronary atherosclerosis? Aldehyde dehydrogenase 2 (ALDH2) is an important enzyme for the oxidation metabolism of aldehyde substances, and it is related to coronary atherosclerosis. The aim of this study is to analyze the relationship between *ALDH2* rs671 polymorphism and the risk of coronary atherosclerosis in T2DM. 701 T2DM patients from January 2019 to February 2024 were retrospectively analyzed, included 364 patients with coronary atherosclerosis [ ≥ 50% coronary stenosis on coronary angiography (CAG)] and 337 controls ( < 50% coronary stenosis). Clinical data collected including gender, age, body mass index (BMI), history of smoking, history of alcohol consumption, hypertension, and lipid level test results. The relationship between *ALDH2* rs671 polymorphism and coronary atherosclerosis risk was analyzed. The frequency of the *ALDH2* rs671 G/G genotype was lower (47.3% vs. 62.9%, *p* < 0.001), whereas the G/A genotype (43.1% vs. 31.8%, *p* = 0.002) and A/A genotype (9.6% vs. 5.3%, *p* = 0.044) were higher in the patients with coronary atherosclerosis than those in the controls. Logistic regression analysis showed that overweight (odds ratio (OR): 1.837, 95% confidence interval (CI): 1.312 –2.573, *p* < 0.001), smoking (OR: 1.644, 95% CI: 1.066–2.535, *p* = 0.025), hypertension (OR: 2.240, 95% CI: 1.607–3.122, *p* < 0.001), dyslipidemia (OR: 1.809, 95% CI: 1.268–2.581, *p* = 0.001), *ALDH2* rs671 G/A or A/A genotype (G/A + A/A vs. G/G, OR: 1.579, 95% CI: 1.131–2.203, *p* = 0.007) were associated with coronary atherosclerosis in T2DM. Overweight, hypertension, dyslipidemia, *ALDH2* rs671 G/A or A/A genotype were associated with coronary atherosclerosis in T2DM patients.

## Introduction

Type 2 diabetes mellitus (T2DM), as a prevalent metabolic disease worldwide, has become a major challenge threatening public health (1, 2). According to data from the International Diabetes Federation (IDF), the number of adult diabetes mellitus patients worldwide exceeds 400 million, and it is showing a rapid growth trend, with T2DM accounting for over 90% of the cases (3, 4). Meanwhile, cardiovascular diseases are the leading cause of death worldwide, accounting for over 30% of all deaths each year (5, 6). It is worth noting that there is an extremely close and complex bidirectional relationship between T2DM and cardiovascular diseases (7). T2DM and cardiovascular diseases do not exist independently but rather promote each other and are causally related in comorbidity (8).

Coronary artery disease (CAD) is a major type of cardiovascular disease, and coronary atherosclerosis serves as the main pathological basis of CAD (9, 10). The pathogenesis of atherosclerosis is complex and involves multiple aspects such as endothelial function impairment (11), enhanced oxidative stress (12), activation of inflammatory responses (13), and lipid deposition (14). In patients with T2DM, high blood glucose can generate advanced glycation end products (AGEs) through non-enzymatic glycation reactions, inducing vascular endothelial cell dysfunction (15). At the same time, insulin resistance leads to an increase in free fatty acid levels, promoting the formation of foam cells and the progression of atherosclerotic plaques (16, 17). Patients with diabetes mellitus have enhanced platelet activity and elevated levels of coagulation factors, while the fibrinolytic system function is weakened. It makes them prone to form blood clots, which can further aggravate coronary artery stenosis or blockage (18). The risk of coronary atherosclerosis in T2DM patients is 2–4 times that of the general population, and the severity of the lesion is more severe and the prognosis is worse (19). Patients with diabetes mellitus and coronary atherosclerosis may exhibit certain peculiarities in their cardiovascular clinical manifestations and lesion characteristics: atypical symptoms, are prone to missed diagnoses, have more complex conditions, and may have a poorer prognosis (20, 21). However, a certain group of T2DM patients are prone to coronary atherosclerosis, while another group of T2DM patients do not suffer from it. Are there any risk factors for T2DM patients to develop coronary atherosclerosis?

Aldehyde dehydrogenase 2 (ALDH2) is an enzyme mainly expressed in mitochondria, and its core function is to catalyze the oxidation metabolism of acetaldehyde and other aldehyde substances (22). Its encoding gene *ALDH2* is located at 12q24.12, and *ALDH2* rs671 (c.1510G > A, p.Glu504Lys) is the most common functional polymorphism (23). The A allele at this site leads to a significant reduction in ALDH2 enzyme activity (only 10–15% of the wild type), causing acetaldehyde to accumulate in the body (24, 25). Current studies have shown that the *ALDH2* rs671 polymorphism is associated with various chronic diseases (26, 27). Some researches have revealed that the reduced activity of ALDH2 can accelerate the process of atherosclerosis through pathways such as enhancing oxidative stress, promoting inflammatory responses, and damaging vascular endothelial function (28–30). Additionally, ALDH2 affect the occurrence and development of T2DM by influencing insulin sensitivity or glucose disposal efficiency (31).

Although the association between the *ALDH2* rs671 polymorphism and T2DM and coronary atherosclerosis has been partially confirmed by some studies, the relationship between *ALDH2* rs671 polymorphism and the risk of coronary artery hardening in T2DM patients remains unclear. Some studies suggest that in the T2DM population, the *ALDH2* rs671 variant may further exacerbate coronary artery damage by superimposing the oxidative stress and acetaldehyde accumulation effects under the high glucose state. However, some studies have not observed a significant association, which may be related to sample size, racial differences, or confounding effects of combined risk factors. Therefore, clarifying the role and mechanism of the *ALDH2* rs671 polymorphism in the occurrence of coronary atherosclerosis in T2DM patients, and providing new biological markers and theoretical basis for the risk prediction and early intervention of coronary atherosclerosis in this population, have important scientific value and clinical significance.

## Materials and methods

### Participants

This study retrospectively collected data on patients with T2DM who were treated at Meizhou People’s Hospital from January 2019 to February 2024. T2DM was defined as blood glucose ≥ 11.1 mmoL/L at any time or fasting blood glucose ≥ 7.0 mmoL/L, or 2-h postprandial plasma glucose level ≥ 11.1 mmoL/L (32). The research group focused on patients with T2DM who also had coronary atherosclerosis. The diagnostic criteria for coronary atherosclerosis: coronary angiography (CAG) showed that the degree of lumen stenosis of at least one coronary artery was ≥ 50% (33). Both cases and controls underwent CAG for similar clinical indications (such as typical chest discomfort, abnormal electrocardiography, or high cardiovascular risk). The inclusion criteria of research group: (1) patients with T2DM; (2) patients with coronary atherosclerosis; and (3) clinical medical records are complete. The control group consisted of patients with T2DM who did not have coronary atherosclerosis. The inclusion criteria of control group: (1) patients with T2DM; (2) patients with < 50% coronary stenosis on CAG, and no history of coronary atherosclerosis, prior percutaneous coronary intervention (PCI) or coronary artery bypass grafting (CABG); and (3) clinical medical records are complete.

**FIGURE 1:**
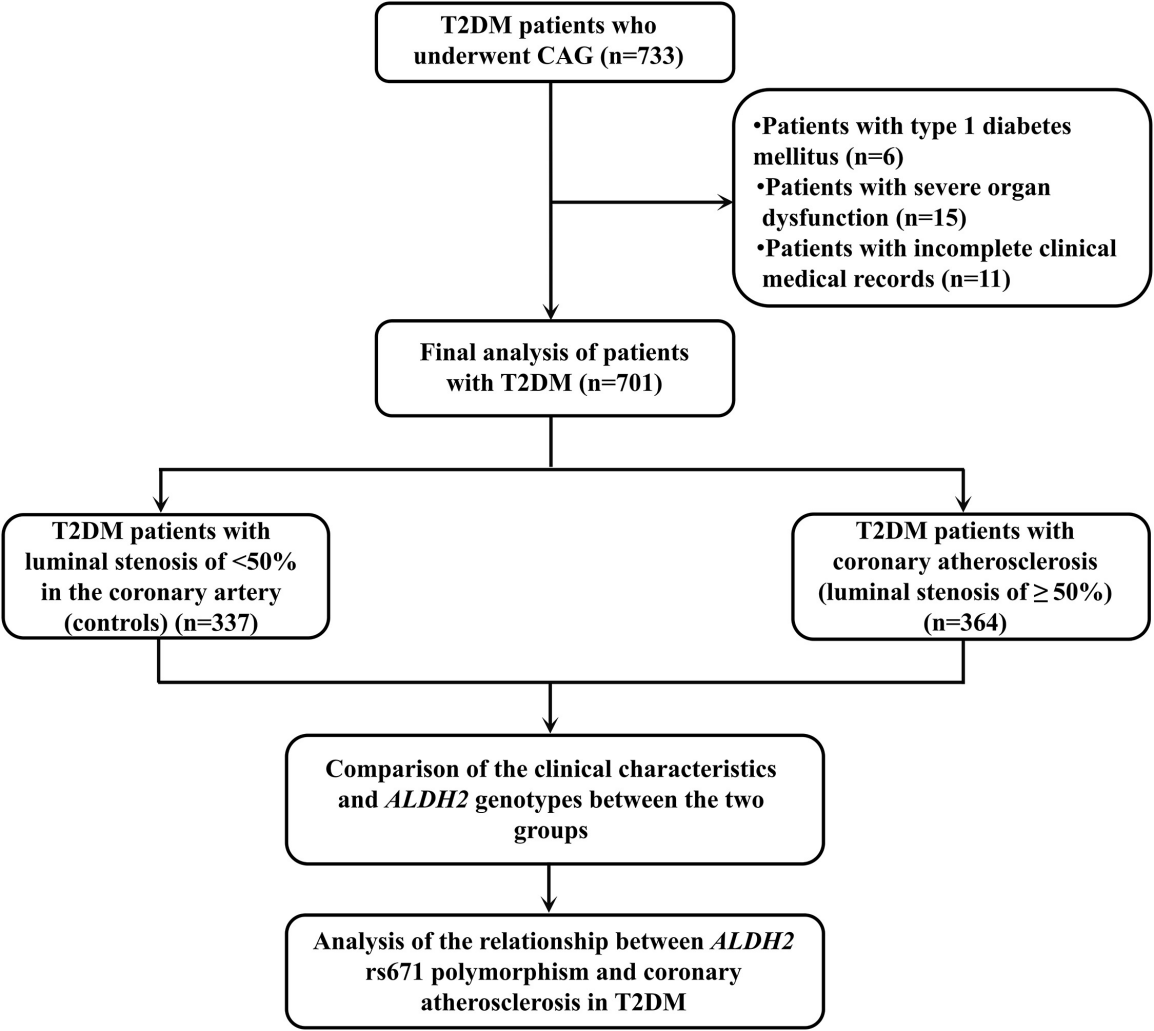
The flowchart of this study.

Patients with the following conditions will be excluded from this study: (1) patients with type 1 diabetes mellitus, special types of diabetes mellitus, and gestational diabetes mellitus; (2) patients with severe organ dysfunction, severe infections, malignant tumors, blood system diseases, and other serious organic diseases; and (3) patients with incomplete clinical medical records. Ultimately, 701 T2DM patients were included in this study, including 364 patients with coronary atherosclerosis (≥ 50% coronary stenosis on CAG) and 337 controls (<50% coronary stenosis). This study was supported by the Ethics Committee of the Meizhou People’s Hospital. The flowchart of this study is shown in Figure 1.

### ALDH2 gene testing

DNA was extracted from the EDTA-anticoagulated whole blood using the genomic DNA extraction kit. *ALDH2* genotyping was conducted using previously described method by detecting the genomic DNA from the subjects included in the study (34, 35). The *ALDH2* rs671 polymorphism has three genotypes: G/G (wild type), G/A (heterozygote), and A/A (mutant homozygote).

### Clinical data collection and stratification of some indicators

Clinical data collected including gender, age, body mass index (BMI), history of cigarette smoking, history of alcohol consumption, hypertension, and laboratory test results [total cholesterol (TC), triglycerides (TG), high-density lipoprotein-cholesterol (HDL-C), low-density lipoprotein-cholesterol (LDL-C)]. We defined elderly patients as aged ≥ 60 years according to widely accepted clinical guidelines and standard geriatric definitions (36, 37). BMI is classified into three categories based on Chinese standards: underweight ( < 18.5 kg/m^2^), normal weight (18.5–23.9 kg/m^2^), and overweight ( ≥ 24.0 kg/m^2^) (38, 39). Hypertension is defined as an average mean systolic blood pressure (SBP) of ≥ 140 mmHg and/or an average mean diastolic blood pressure (DBP) of ≥ 90 mmHg (40). Participants with a hypertension history were classified as those who had been formally diagnosed with hypertension in the past or were currently receiving oral antihypertensive therapy. A smoking history was assigned to individuals who smoked at least one cigarette daily for 1 year or longer, or who had quit smoking for < 6 months. Furthermore, a history of alcohol consumption was defined as regular alcohol intake, specifically drinking alcoholic beverages at least once per week for ≥ 6 consecutive months. The criteria for dyslipidemia: (1) TC ≥ 6.22 mmoL/L, (2) TG ≥ 2.26 mmoL/L, or (3) LDL-C ≥ 4.14 mmoL/L, according to the Guidelines for the Prevention and Control of Dyslipidemia in Chinese Adults (41, 42).

### Statistical analysis

The statistical analyses of this study were conducted using SPSS 26.0 software. All continuous variables were subjected to normality test. Continuous variables conforming to a normal distribution were expressed as mean ± standard deviation (SD) and compared using the independent-samples *t*-test or one-way analysis of variance (ANOVA). Continuous variables that do not follow a normal distribution were expressed as median and interquartile range (IQR) (25–75th percentiles), and conducted using Mann-Whitney U test for groups comparisons. The χ^2^ test was used to compare the genotypes and alleles frequencies among different groups, and to evaluate the Hardy-Weinberg equilibrium in the research group and control group. Logistic regression analysis was applied to examine the relationship of *ALDH2* genotypes and coronary atherosclerosis risk. As age, BMI, history of cigarette smoking, alcohol consumption, hypertension, and dyslipidemia are all traditional risk factors associated with cardiovascular disease, all these variables were forcibly included in the multivariate analysis in this study. All statistical tests were two-sided, and a *p* < 0.05 was considered statistically significant.

## Results

### Comparison of clinical features of patients with and without coronary atherosclerosis in T2DM patients

In this study, there were 504 (71.9%) male and 197 (28.1%) female patients. A total of 520 (74.2%) patients were aged ≥ 60 years, and 297 (42.4%) were overweight. Among the patients with T2DM, 182 (26.0%) had a smoking history, 87 (12.4%) had a history of alcohol consumption, and 369 (52.6%) had hypertension. The mean levels of TC, TG, HDL-C, and LDL-C was 4.25 (3.42, 5.19) mmoL/L, 1.55 (1.10, 2.30) mmoL/L, 1.03 (0.80, 1.25) mmoL/L, and 2.35 (1.78, 3.03) mmoL/L, respectively. There were 220 (31.4%) patients with dyslipidemia (Table 1).

**TABLE 1:** Comparison of clinical features of patients with and without coronary atherosclerosis in T2DM patients.

Variables	All patients (*n* = 701)	Controls (*n* = 337)	Patients with coronary atherosclerosis (*n* = 364)	*p* (χ ^2^)
Gender				
Male, n(%)	504 (71.9%)	241 (71.5%)	263 (72.3%)	0.867 (χ^2^ = 0.047)
Female, n(%)	197 (28.1%)	96 (28.5%)	101 (27.7%)	
Age (years)				
< 60, n(%)	181 (25.8%)	98 (29.1%)	83 (22.8%)	0.070 (χ^2^ = 3.601)
≥ 60, n(%)	520 (74.2%)	239 (70.9%)	281 (77.2%)	
BMI (kg/m^2^)				
Underweight, n (%)	41 (5.8%)	27 (8.0%)	14 (3.8%)	< 0.001 (χ^2^ = 26.856)
Normal weight, n (%)	363 (51.8%)	200 (59.3%)	163 (44.8%)	
Overweight, n (%)	297 (42.4%)	110 (32.6%)	187 (51.4%)	
Cigarette smoking				
No, n(%)	519 (74.0%)	251 (74.5%)	268 (73.6%)	0.863 (χ^2^ = 0.066)
Yes, n(%)	182 (26.0%)	86 (25.5%)	96 (26.4%)	
Alcohol consumption				
No, n(%)	614 (87.6%)	269 (79.8%)	345 (94.8%)	< 0.001 (χ^2^ = 36.018)
Yes, n(%)	87 (12.4%)	68 (20.2%)	19 (5.2%)	
Hypertension				
No, n(%)	332 (47.4%)	200 (59.3%)	132 (36.3%)	< 0.001 (χ^2^ = 37.401)
Yes, n(%)	369 (52.6%)	137 (40.7%)	232 (63.7%)	
Levels of serum lipid				
TC, mmoL/L, median (IQR)	4.25 (3.42, 5.19)	4.02 (3.28, 4.90)	4.49 (3.60, 5.52)	< 0.001 (*Z* = -4.620)
TG, mmoL/L, median (IQR)	1.55 (1.10, 2.30)	1.44 (1.04, 2.01)	1.66 (1.16, 2.39)	< 0.001 (*Z* = -3.803)
HDL-C, mmoL/L, median (IQR)	1.03 (0.80, 1.25)	0.97 (0.70, 1.25)	1.07 (0.90, 1.26)	< 0.001 (*Z* = -4.091)
LDL-C, mmoL/L, median (IQR)	2.35 (1.78, 3.03)	2.22 (1.71, 2.78)	2.50 (1.87, 3.23)	< 0.001 (*Z* = -4.045)
Dyslipidemia				
No, n(%)	481 (68.6%)	253 (75.1%)	228 (62.6%)	< 0.001 (χ^2^ = 12.569)
Yes, n(%)	220 (31.4%)	84 (24.9%)	136 (37.4%)	

T2DM, type 2 diabetes mellitus; BMI, body mass index; TC, total cholesterol; TG, triglycerides; HDL-C, high-density lipoprotein-cholesterol; LDL-C, low-density lipoprotein-cholesterol; IQR, interquartile range.

There was significant difference in the distribution of BMI (χ^2^ = 26.856, *p* < 0.001) between patients with coronary atherosclerosis and controls. The proportion of history of alcohol consumption (5.2% vs. 20.2%, *p* < 0.001) was lower, while hypertension (63.7% vs. 40.7%, *p* < 0.001) and dyslipidemia (37.4% vs. 24.9%, *p* < 0.001) in patients with coronary atherosclerosis was higher than those in controls, respectively (Table 1).

### Distribution frequencies of *ALDH2* rs671 genotypes and alleles in patients with coronary atherosclerosis and controls

In total, 384 (54.8%), 264 (37.7%), and 53 (7.6%) subjects carried the ALDH2 rs671 G/G, G/A, and A/A genotypes, respectively. The *ALDH2* rs671 genotypes in the patients with coronary atherosclerosis (χ^2^ = 0.009, *p* = 0.924), and controls (χ^2^ = 0.851, *p* = 0.356) conformed to the Hardy-Weinberg equilibrium, respectively. Compared with controls, patients with coronary atherosclerosis exhibited a lower frequency of the *ALDH2* rs671 G/G genotype (47.3% vs. 62.9%, *p* < 0.001), but higher frequencies of the G/A genotype (43.1% vs. 31.8%, *p* = 0.002) and A/A genotype (9.6% vs. 5.3%, *p* = 0.044). The frequency of the *ALDH2* rs671 G allele was lower (68.8% vs. 78.8%) and *ALDH2* rs671 A allele was higher (31.2% vs. 21.2%) in the patients with coronary atherosclerosis than in controls (χ^2^ = 17.889, *p* < 0.001) (Table 2).

**TABLE 2:** Distribution frequencies of *ALDH2* rs671 genotype and allele in patients with and without coronary atherosclerosis.

Variables	Genotype/allele	All patients (*n* = 701)	Controls (*n* = 337)	Patients with coronary atherosclerosis (*n* = 364)	*p* (χ ^2^)
*ALDH2* rs671 genotypes					
	G/G	384 (54.8%)	212 (62.9%)	172 (47.3%)	< 0.001 (χ^2^ = 17.313)
	G/A	264 (37.7%)	107 (31.8%)	157 (43.1%)	0.002 (χ^2^ = 9.655)
	A/A	53 (7.6%)	18 (5.3%)	35 (9.6%)	0.044(χ^2^ = 4.574)
*ALDH2* rs671 alleles					
	G	1,032 (73.6%)	531 (78.8%)	501 (68.8%)	< 0.001 (χ^2^ = 17.889)
	A	370 (26.4%)	143 (21.2%)	227 (31.2%)	
	HWE (χ^2^, *P*)	χ^2^ = 0.660, *p* = 0.417	χ^2^ = 0.851, *p* = 0.356	χ^2^ = 0.009, *p* = 0.924	

ALDH2, aldehyde dehydrogenase 2; HWE, Hardy Weinberg Equilibrium.

### Comparison of clinical characteristics of cases with different *ALDH2* rs671 genotypes

There were significant differences in the proportions of individuals with cigarette smoking, and alcohol consumption among patients with *ALDH2* rs671 G/G genotype and patients with *ALDH2* rs671 G/A or A/A genotypes. There was no significant difference in the distribution of gender, BMI, hypertension, and dyslipidemia in patients with *ALDH2* rs671 G/G genotype and patients with *ALDH2* rs671 G/A or A/A genotypes (Table 3).

**TABLE 3:** Comparison of clinical characteristics of cases with different *ALDH2* rs671 genotypes.

Variables	G/G (*n* = 384)	G/A or A/A (*n* = 317)	*p* (χ ^2^)
Gender			
Male, n(%)	285 (74.2%)	219 (69.1%)	0.151 (χ^2^ = 2.265)
Female, n(%)	99 (25.8%)	98 (30.9%)	
Age (years)			
< 60, n(%)	119 (31.0%)	62 (19.6%)	0.001 (χ^2^ = 11.847)
≥ 60, n(%)	265 (69.0%)	255 (80.4%)	
BMI (kg/m^2^)			
Underweight, n (%)	23 (6.0%)	18 (5.7%)	0.327 (χ^2^ = 2.238)
Normal weight, n (%)	208 (54.2%)	155 (48.9%)	
Overweight, n (%)	153 (39.8%)	144 (45.4%)	
Cigarette smoking			
No, n(%)	270 (70.3%)	249 (78.5%)	0.015 (χ^2^ = 6.128)
Yes, n(%)	114 (29.7%)	68 (21.5%)	
Alcohol consumption			
No, n(%)	304 (79.2%)	310 (97.8%)	< 0.001 (χ^2^ = 55.414)
Yes, n(%)	80 (20.8%)	7 (2.2%)	
Hypertension			
No, n(%)	188 (49.0%)	144 (45.4%)	0.363 (χ^2^ = 0.869)
Yes, n(%)	196 (51.0%)	173 (54.6%)	
Levels of serum lipid			
TC, mmoL/L, median (IQR)	4.18 (3.37, 5.20)	4.32 (3.51, 5.18)	0.367 (*Z* = -0.902)
TG, mmoL/L, median (IQR)	1.64 (1.18, 2.39)	1.48 (1.08, 2.16)	0.020 (*Z* = -2.333)
HDL-C, mmoL/L, median (IQR)	1.01 (0.76, 1.20)	1.06 (0.85, 1.31)	0.005 (*Z* = -2.795)
LDL-C, mmoL/L, median (IQR)	2.30 (1.75, 3.00)	2.37 (1.80, 3.06)	0.421 (*Z* = -0.805)
Dyslipidemia			
No, n(%)	256 (66.7%)	225 (71.0%)	0.252 (χ^2^ = 1.499)
Yes, n(%)	128 (33.3%)	92 (29.0%)	

T2DM, type 2 diabetes mellitus; BMI, body mass index.

### Logistic regression analysis of relationship of *ALDH2* rs671 genotypes and coronary atherosclerosis in patients with T2DM

We analyzed the interaction between alcohol consumption and the *ALDH2* rs671 genotypes on coronary atherosclerosis, and the results showed that the interaction between the *ALDH2* rs671 genotypes and alcohol consumption was not statistically significant (Table 4). Univariate logistic regression analysis showed that overweight [odds ratio (OR): 2.086, 95% confidence interval (CI): 1.525–2.854, *p* < 0.001], hypertension (OR: 2.566, 95% CI: 1.892–3.480, *p* < 0.001), dyslipidemia (OR: 1.797, 95% CI: 1.297–2.488, *p* < 0.001), *ALDH2* rs671 G/A genotype (G/A vs. G/G, OR: 1.809, 95% CI: 1.317–2.484, *p* < 0.001) and A/A genotype (A/A vs. G/G, OR: 2.397, 95% CI: 1.311–4.380, *p* = 0.004) were significantly associated with coronary atherosclerosis in T2DM patients. Multivariate logistic regression analysis without alcohol consumption adjustment showed that overweight (OR: 1.890, 95% CI: 1.359–2.628, *p* < 0.001), hypertension (OR: 2.236, 95% CI: 1.615–3.095, *p* < 0.001), dyslipidemia (OR: 1.788, 95% CI: 1.262–2.532, *p* = 0.001), *ALDH2* rs671 G/A or A/A genotype (G/A + A/A vs. G/G, OR: 1.943, 95% CI: 1.407–2.681, *p* < 0.001) were associated with coronary atherosclerosis in T2DM. And multivariate logistic regression analysis with alcohol consumption adjustment showed that *ALDH2* rs671 G/A or A/A genotype (G/A + A/A vs. G/G, OR: 1.579, 95% CI: 1.131–2.203, *p* = 0.007) also associated with coronary atherosclerosis in T2DM (Table 5).

**TABLE 4:** Association analysis of the interaction between alcohol consumption and *ALDH2* rs671 genotypes with coronary atherosclerosis.

Alcohol consumption	*ALDH2* rs671 genotypes	OR (95% CI)	*P-*values
No	G/G	1.000 (reference)	
No	G/A + A/A	1.368 (0.993–1.883)	0.055
Yes	G/G	0.717 (0.094–1.334)	0.071
Yes	G/A + A/A	5.472 (0.651–45.994)	0.118

OR, odds ratio; CI, confidence interval.

**TABLE 5:** Logistic regression analysis of relationship of *ALDH2* rs671 genotypes and coronary atherosclerosis in patients with T2DM.

Variables	Univariate OR (95% CI)	*P-*values	Multivariate OR (without alcohol adjustment) (95% CI)	*P-*values	Multivariate OR (with alcohol adjustment) (95% CI)	*P-*values
Gender (male vs. female)	1.037 (0.746–1.442)	0.828	1.296 (0.882–1.905)	0.187	1.407 (0.956–2.072)	0.084
Age ( ≥ 60 vs. < 60 years old)	1.388 (0.989–1.949)	0.058	1.192 (0.819–1.734)	0.360	1.155 (0.785–1.699)	0.466
BMI (kg/m^2^)						
Normal weight	1.000 (reference)	-	1.000 (reference)	-	1.000 (reference)	-
Underweight	0.636 (0.323–1.253)	0.191	0.784 (0.387–1.586)	0.498	0.941 (0.454–1.950)	0.871
Overweight	2.086 (1.525–2.854)	< 0.001	1.890 (1.359–2.628)	< 0.001	1.837 (1.312–2.573)	< 0.001
Cigarette smoking (yes vs. no)	1.045 (0.746–1.466)	0.797	1.129 (0.765–1.665)	0.541	1.644 (1.066–2.535)	0.025
Hypertension (yes vs. no)	2.566 (1.892–3.480)	< 0.001	2.236 (1.615–3.095)	< 0.001	2.240 (1.607–3.122)	< 0.001
Dyslipidemia (yes vs. no)	1.797 (1.297–2.488)	< 0.001	1.788 (1.262–2.532)	0.001	1.809 (1.268–2.581)	0.001
*ALDH2* rs671 genotypes						
G/G	1.000 (reference)	-	1.000 (Reference)	-	1.000 (Reference)	-
G/A	1.809 (1.317–2.484)	< 0.001	1.829 (1.303–2.566)	< 0.001	1.492 (1.052–2.116)	0.025
A/A	2.397 (1.311–4.380)	0.004	2.627 (1.398–4.937)	0.003	2.094 (1.108–3.958)	0.023
G/A + A/A	1.893 (1.400–2.561)	< 0.001	1.943 (1.407–2.681)	< 0.001	1.579 (1.131–2.203)	0.007

T2DM, type 2 diabetes mellitus; BMI, body mass index; OR, odds ratio; CI, confidence interval.

To further ensure the robustness of our results, we also performed sensitivity analysis using different thresholds of age (such as ≥ 50 and < 50 years old, ≥ 55 and < 55 years old, ≥ 60 and < 60 years old, ≥ 65 and < 65 years old). The main conclusion remained unchanged: *ALDH2* rs671 G/A or A/A genotype was significantly associated with coronary atherosclerosis in T2DM, supporting the stability of our findings (Table 6).

**TABLE 6:** Logistic regression analysis of the association of *ALDH2* rs671 genotypes with coronary atherosclerosis in patients with T2DM, adjusted for age across different thresholds.

Variables	Adjusted β /OR (without alcohol adjustment) (95% CI)	*P-*values	Adjusted β /OR (with alcohol adjustment) (95% CI)	*P-*values
Age ( ≥ 50 and < 50 years old)				
*ALDH2* rs671 genotypes (G/A + A/A vs. G/G)	1.962 (1.421–2.707)	< 0.001	1.592 (1.140–2.221)	0.006
Age ( ≥ 55 and < 55 years old)				
*ALDH2* rs671 genotypes (G/A + A/A vs. G/G)	2.014 (1.459–2.779)	< 0.001	1.627 (1.166–2.270)	0.004
Age ( ≥ 60 and < 60 years old)				
*ALDH2* rs671 genotypes (G/A + A/A vs. G/G)	1.943 (1.407–2.681)	< 0.001	1.579 (1.131–2.203)	0.007
Age ( ≥ 65 and < 65 years old)				
*ALDH2* rs671 genotypes (G/A + A/A vs. G/G)	1.912 (1.385–2.640)	< 0.001	1.549 (1.109–2.164)	0.010

OR, odds ratio; CI, confidence interval. Adjust for: gender, age, BMI, cigarette smoking, hypertension, and dyslipidemia.

## Discussion

Even if patients with T2DM do not have obvious symptoms of coronary heart disease, their coronary arteries may still have varying degrees of atherosclerotic lesions (43). Moreover, the atherosclerosis of the coronary arteries in T2DM patients often presents with multi-vessel involvement and increased plaque instability, and poorer prognosis (20, 21). What are the risk factors for coronary atherosclerosis in the T2DM population? This study investigated the relationship between the *ALDH2* rs671 polymorphism and the risk of coronary atherosclerosis in patients with T2DM. The results show that overweight, hypertension, dyslipidemia, *ALDH2* rs671 G/A or A/A genotype were associated with coronary atherosclerosis in T2DM patients.

*ALDH2* rs671 polymorphism significantly reduces the catalytic activity of ALDH2, leading to a decline in the metabolic capacity of acetaldehyde (44). In patients with T2DM, this variant may affect the occurrence and development of coronary atherosclerosis through multiple pathways. On one hand, the reduced ALDH2 activity leads to the accumulation of acetaldehyde in the body, and acetaldehyde can induce oxidative stress responses, promote vascular endothelial cell damage, and disrupt the function of the vascular endothelial barrier, creating conditions for the initiation of atherosclerosis (45). On the other hand, studies have shown that ALDH2 is also involved in the clearance of oxidative stress products (such as 4-hydroxynonenal) in the body (46, 47). The decreased enzyme activity caused by *ALDH2* rs671 polymorphism weakens the body’s ability to clear lipid peroxidation products, aggravates lipid deposition and inflammatory responses in the vessel wall, and accelerates the formation of atherosclerotic plaques. In patients with T2DM, there are already enhanced oxidative stress and chronic inflammatory conditions (48). The *ALDH2* rs671 polymorphism may exacerbate these pathological processes, thereby increasing the risk of coronary atherosclerosis.

Meanwhile, the metabolic abnormalities such as insulin resistance, hyperglycemia, and lipid disorders that exist in patients with T2DM may have a synergistic effect with the *ALDH2* gene rs671 polymorphism. High blood glucose level can trigger a series of pathological processes such as activation of the polyol pathway and accumulation of advanced glycation end products, further exacerbating oxidative stress and vascular endothelial damage (49, 50). The abnormal function of ALDH2 may not be able to effectively counteract these damaging factors, making coronary artery vessels more vulnerable and increasing the risk of atherosclerosis. Additionally, some studies suggest that the *ALDH2* rs671 polymorphism may affect platelet function (51) and the balance of the coagulation system (52), potentially further promoting thrombosis and worsening the degree of coronary artery stenosis in patients with T2DM.

The relationship between the *ALDH2* rs671 polymorphism and coronary artery disease has been reported previously. *ALDH2* rs671 was associated with coronary artery disease risk (26, 53–56). The *ALDH2* rs671 A allele is significantly associated with complications in diabetic patients undergoing percutaneous coronary intervention (PCI) (57). On the contrary, some studies suggested that the *ALDH2* rs671 polymorphism was not associated with the severity of coronary atherosclerosis (58), postoperative complications (59). He et al. demonstrated that the *ALDH2* rs671 GA/AA genotype was a risk factor for cardio-cerebrovascular complications in patients with T2DM (60). Some studies found that the *ALDH2* rs671 AA genotype was an independent risk factor for coronary artery stenosis (61) and coronary artery disease (CAD) (62, 63) in elderly patients with T2DM. Except for the elderly patients with T2DM, the results of this study indicated that the *ALDH2* rs671 polymorphism was associated with the risk of coronary atherosclerosis in unselected patients with T2DM. This study is one of the few investigations on the relationship between *ALDH2* rs671 polymorphism and the risk of coronary atherosclerosis in patients with T2DM.

In addition, the proportion of alcohol consumption among T2DM patients with the *ALDH2* G/G genotype was notably higher than that in those carrying the G/A and A/A genotypes in the current study. Individuals harboring the *ALDH2* G/A and A/A genotypes present relatively low ALDH2 enzymatic activity, and various adverse reactions occur rapidly even after light alcohol intake. Given their impaired ability to metabolize and clear acetaldehyde, subjects with the *ALDH2* G/A and A/A genotypes tend to voluntarily decrease their ethanol intake (64, 65). The *ALDH2* rs671 A allele exhibits a dual effect of bidirectional regulation in the occurrence and development of cardiovascular diseases. At the biological mechanism, this allele is associated with aldehyde metabolism and oxidative stress injury, thereby promoting the occurrence and progression of atherosclerosis. From the perspective of behavioral phenotype, the individuals carrying the *ALDH2* G/A or A/A genotype tend to actively reduce ethanol intake. Therefore, the *ALDH2* rs671 A allele may reduce the risk of cardiovascular diseases by altering alcohol-related behaviors and effectively decreasing alcohol intake. However, our analysis demonstrated that the A allele was independently associated with an increased risk of coronary atherosclerosis (OR = 1.579). This apparent discrepancy can be explained by the fact that the detrimental biological effects of ALDH2 deficiency, including excessive aldehyde accumulation, oxidative stress, and endothelial dysfunction, far outweigh the modest beneficial impact of decreased alcohol consumption in our study population. Consequently, the net effect of carrying the A allele is a pro-atherogenic phenotype and an overall increase in cardiovascular risk. These findings highlight the dominant role of genetic-mediated metabolic disturbances over behavioral changes in the pathogenesis of coronary atherosclerosis. The coexistence of such dual effects mediated by this genetic variant suggests that the final net effect of the *ALDH2* rs671 A allele on cardiovascular health depends on the comprehensive interplay between biological injury pathways and behavioral protective pathways, which provides a key perspective for elucidating the pathogenesis of cardiovascular diseases modulated by the interaction between genetic factors and lifestyle.

Regarding the interaction between *ALDH2* genotypes and alcohol consumption, the interaction *p-*value was 0.055, which did not reach the conventional threshold for statistical significance. However, we observed a marked increase in the genotype-associated odds ratio from 1.368 in non-drinkers to 5.472 in drinkers. This pattern suggests a potential trend toward effect modification, in which alcohol consumption may exacerbate the genetic risk related to ALDH2 deficiency. Such a trend is biologically plausible given the pathophysiological role of ALDH2 in alcohol metabolism and aldehyde accumulation. Although the interaction was not statistically significant in the present study, the limited sample size and relatively low statistical power within subgroups may have precluded the detection of a significant interaction. This observation warrants further validation in larger, well-powered cohorts.

It is worth noting that smoking was not significant in the univariate analysis but was significant in the multivariate analysis (with alcohol adjustment). The inconsistent significance of the smoking variable in univariate and multivariate analyses is a common phenomenon in clinical and epidemiological regression models, which is mainly caused by the adjustment of confounding factors. In the univariate analysis, the association between smoking and coronary atherosclerosis was masked by potential confounding effects. After adjusting for other relevant covariates (with alcohol adjustment), the independent effect of smoking on coronary atherosclerosis was identified, leading to statistically significant results in the multivariate analysis. This result indicates that smoking may exert an independent effect only after the interference of confounding factors has been excluded.

This study has certain limitations. Firstly, although the samples included in this study cover multiple age and gender groups, as a single-center retrospective study, the subjects were only derived from a single medical institution with a relatively small overall sample size, which may lead to selection bias and limit the generalizability and universal applicability of the results. Larger-scale, multi-center, long-term follow-up cohort studies are needed to further verify the generalizability of the results of this study. Secondly, this study only isolatedly explored the role of the *ALDH2* rs671 polymorphism, and did not construct a multi-factor interaction model. In fact, the activity of the ALDH2 encoded by *ALDH2* gene is significantly regulated by alcohol intake (66). Individuals carrying *ALDH2* rs671 polymorphism will experience ethanol accumulation after drinking, and ethanol may have a synergistic damaging effect with diabetes-induced high sugar environment. At the same time, metabolic disorders caused by high-fat and high-sugar dietary habits, may all affect the expression of the *ALDH2* gene through epigenetic modifications (67). Future research should design case-control matching trials to systematically evaluate the interaction network of gene-environment factors. Moreover, this study mainly relies on clinical data mining and lacks direct evidence at the molecular mechanism level. Although some studies have shown that ALDH2 participates in regulating vascular endothelial cell function and inflammatory responses, how the *ALDH2* rs671 variant affects downstream signaling pathways by influencing enzyme activity, and thereby promotes the formation of atherosclerotic plaques, still requires functional validation experiments using cell models and animal models. In particular, it is necessary to systematically analyze the molecular network that regulates atherosclerosis through metabolomics, proteomics and other multi-omics techniques.

Future research can be conducted in the following directions. First, expand the sample size and carry out cross-ethnic multi-center studies to clarify the universality of the association between the *ALDH2* rs671 polymorphism and the risk of coronary atherosclerosis in patients with T2DM. Second, through molecular biological experiments, deeply explore the specific mechanisms by which the *ALDH2* rs671 affects vascular endothelial function, lipid metabolism, and inflammatory response, providing theoretical basis for the development of targeted intervention drugs. Third, analyze the interaction between this polymorphism and environmental factors, and formulate individualized cardiovascular disease prevention strategies for patients with T2DM. Fourth, combine imaging and biomarkers to evaluate the impact of the *ALDH2* polymorphisms on the progression and prognosis of coronary atherosclerosis, providing reference for clinical risk stratification and treatment decisions. Overall, in-depth study of the association and mechanism between the two is expected to provide new ideas and targets for the precise prevention and treatment of cardiovascular complications in patients with T2DM.

## Conclusion

In summary, overweight, hypertension, dyslipidemia, *ALDH2* rs671 G/A or A/A genotype were associated with coronary atherosclerosis in T2DM patients. It means that T2DM patients with *ALDH2* rs671 G/A or A/A genotype and some traditional risk factors (overweight, smoking, hypertension, and dyslipidemia) need to be aware of the risk of coronary atherosclerosis.

## Data Availability

The original contributions presented in the study are included in the article/supplementary material, further inquiries can be directed to the corresponding author.
